# Factors predicting the outcome of patients admitted with COVID-19 infection at Pietersburg Hospital, Polokwane, Limpopo province

**DOI:** 10.4102/sajid.v41i1.782

**Published:** 2026-02-18

**Authors:** Koena J. Moabelo, Phetho Mangena, Musa E. Setati, Mokibe J. Nchabeleng

**Affiliations:** 1 Department of Internal Medicine, School of Medicine, University of Limpopo, Polokwane, South Africa; 2 Department of Public Health Medicine, School of Medicine, University of Limpopo, Polokwane, South Africa; 3 Department of Paediatrics and Child Health, School of Medicine, University of Limpopo, Polokwane, South Africa

**Keywords:** SARS-CoV-2, COVID-19, outcomes, comorbidities, pandemic

## Abstract

**Background:**

The emergence of the novel coronavirus disease 2019 (COVID-19), in late 2019 in Wuhan City, has led to a global outbreak of COVID-19, culminating in the declaration of a pandemic by the World Health Organization.

**Objectives:**

The aim of the study was to evaluate the factors predicting the outcome in patients with COVID-19 infection.

**Method:**

This was a retrospective study using secondary data of patients admitted with laboratory-confirmed severe acute respiratory syndrome coronavirus 2 (SARS-CoV-2) polymerase chain reaction (PCR) results at Pietersburg Hospital between 01 March 2020 and 31 March 2021, which were extracted from DATCOV portal.

**Results:**

There were 444 eligible study participants, of which 225 (50.7%) were female and 219 (49.3%) were male patients. Their median age was 57 years. A total of 159 (36%) in-hospital deaths related to COVID-19 infection were reported. Using the logistic regression model, two predictor variables, age and intensive care unit (ICU) admission were independently associated with mortality. An increase in patient’s age per year, increased the odds of dying. The ICU admission had a three-fold odds for non-survival. In-hospital mortality was associated with shorter length of hospitalisation because of disease severity and late patient presentation, with a median duration of 4 days (interquartile range [IQR] 2.0–9.0).

**Conclusion:**

Age and ICU admission were significantly related to non-survival, with hypertension being the common factor in all deaths reported.

**Contribution:**

The study emphasises the importance of pandemic preparedness and strengthening healthcare services to protect vulnerable groups such as the elderly population.

## Introduction

In December 2019, the new coronavirus outbreak caused by severe acute respiratory syndrome coronavirus 2 (SARS-CoV-2) infection was classified as a major global health threat.^1^ The novel coronavirus causes a severe acute respiratory syndrome that typically manifests with acute respiratory symptoms such as cough and dyspnoea, joint aches, fatigue, myalgia, and flu-like symptoms. Asymptomatic cases have also been documented.^2,3^ Because of various waves experienced, coronavirus disease 2019 (COVID-19) cases reported globally have breached over 600 million cases with approximately 6.5 million fatalities. Various studies have indicated that pre-existing comorbidities and older age were associated with deleterious effect in COVID-19 patients.^2,4^ It has been shown that in some patients, COVID-19 infection aggravated co-existing disorders through the host immune response and frequently revealed underlying diseases with rapid progression.^3^

The effects of comorbidities on a variety of diseases have been extensively studied globally.^4,5^ People with underlying health difficulties have severe, life-threatening sickness and significant mortality, according to a systematic review and meta-analysis on the frequency of comorbidities in the Middle East Respiratory Syndrome coronavirus (MERS-CoV).^6^

Non-communicable diseases (NCDs) such as hypertension, type 2 diabetes, obesity and being overweight, are prevalent and contribute to the burden of disease in South Africa.^7^ Chronic comorbid conditions are typically difficult to control for many reasons, including an unorganised healthcare system, disruptions in the supply of medications and a lack of health professional skills and ability.^8^ Therefore, prevention and control of comorbidities are crucial to avert the risk of severe COVID-19 illness and death.^7^

Most hospitalised COVID-19 infected patients required respiratory care because of pneumonia. Those with comorbidities developed severe form of COVID-19 disease that necessitated high or intensive care unit (ICU) admission.

The primary cause of COVID-19 admissions at Pietersburg hospital was pneumonia with many having concomitant conditions. The goal of the study was to investigate factors that predicted the outcome of patients with COVID-19 infection and any associated negative consequences at Pietersburg Hospital.

## Research methods and design

### Study design and setting

The study was performed from 01 March 2020 until 31 March 2021 at Pietersburg Hospital, a tertiary centre in Polokwane. It was a retrospective study using secondary data accessed from DATCOV portal developed by National Institute for Communicable Diseases (NICD).

### Study population

#### Inclusion criteria

Population group enrolled included all adults and adolescents outside paediatric age group (i.e. > 12 years old) with a confirmed severe acute respiratory syndrome coronavirus 2 (SARS-CoV-2) polymerase chain reaction (PCR) test admitted to our isolation wards and ICU.

#### Exclusion criteria

Persons under investigation (PUI) without a laboratory confirmed test but suspected to have COVID-19 infection based on symptoms and signs while awaiting SARS-CoV-2 PCR results were excluded.

### Data collection

Secondary data were extracted from the NICD DATCOV portal, which is a web-based disease surveillance system (version v2.4.35). The NICD approved the data collection prior to interrogating the data. The following variables were extracted: demographics, comorbidities (self-reported, based on clinical diagnosis, and/or on treatment), length of hospitalisation and patient outcomes (discharged alive, transferred to other facility, died).

### Data analysis

The data were analysed with Spotfire Statistica 14.2.0 (TIBCO, Cloud Software Group Inc, United States). The median with interquartile ranges (IQR) was used to present continuous variables. Cross-tabulations, percentages, and numbers were used to present categorical variables. The Chi-square test was employed to ascertain whether categorical variables were associated. Mann–Whitney U-test was used to compare the continuous variables. Univariate analysis was used to identify variables that were significant. Using a multivariable logistic regression model, the link between risk factors identified through univariate analysis and outcome was examined. In this study, COVID-19 survival and in-hospital death were the endpoints. The odds ratio (OR) and 95% confidence interval (CI) of the chosen predictor variables were used to express the results. A *p*-value below 0.05 was considered statistically significant.

### Ethical considerations

Ethical approval was granted by the Turfloop Research Ethics Committee (TREC/99/2022:PG) of the University of Limpopo, the Limpopo Department of Health (Ref: LP_2022-05-029), and the Polokwane-Mankweng Research Ethics Committee (PMREC 31 August UL 2022/B). The data collected was completely anonymised and no patient identifying details were documented. Because data collected was from a secondary source, application for waiver of informed consent was made as per the institutional ethics committee guidelines.

## Results

**FIGURE 1:**
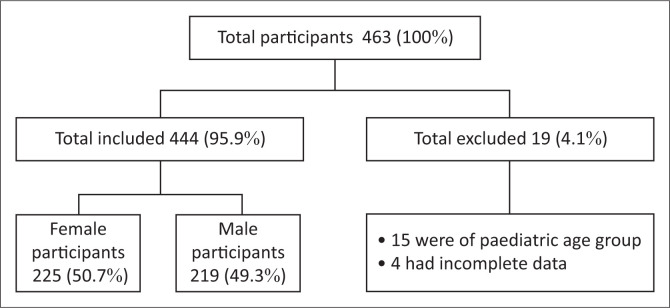
Study participants who met inclusion criteria (*N* = 444).

During the period of the study, a total of 463 patients with confirmed COVID-19 infection were admitted at Pietersburg hospital. Nineteen patients were excluded for not meeting the inclusion criteria. The study sample comprised 225 (50.7%) female and 219 (49.3%) male participants, with a median age of 57 (13–96) years (Figure 1).

**FIGURE 2:**
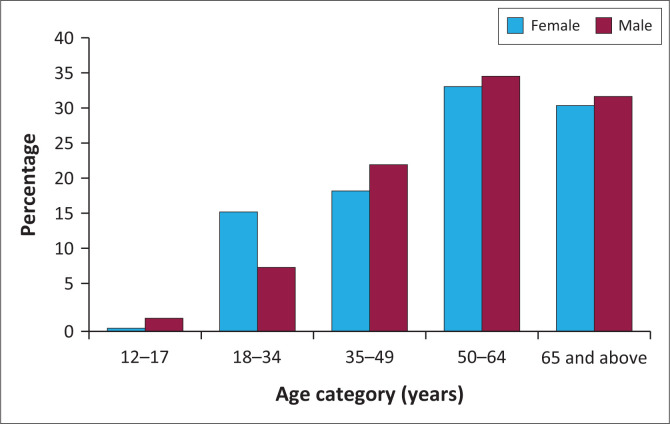
Age groups of patients with COVID-19 infection seen in the study (*N* = 444).

Most hospitalised patients were aged between 50 years and 64 years (34.0%). The hospitalised adolescent patients were not severely affected. The adolescent group falling within the age category of 12–17 years only contributed 1.1% of the disease versus 67.6% of the adult (18–64 years) and 31.3% of the elderly groups (65 years and above) (Figure 2).

Of the 444 patients, 311 (70%) had comorbidities, namely, hypertension (*n* = 208 [51%]), diabetes (*n* = 152 [38%]), human immunodeficiency virus (HIV) (*n* = 54 [24%]), chronic renal failure (*n* = 32 [8.5%]), cardiac disease (*n* = 27 [7%]), asthma (*n* = 20 [5%]), chronic obstructive pulmonary disease (COPD) (*n* = 14 [3.8%]), malignancy (*n* = 12 [3%]) and active tuberculosis (TB) (*n* = 5 [1.5%]) (Table 1).

**TABLE 1:** Comorbidities present in patients with COVID-19.

Comorbidities	Female			Male		
	*n*	*N*	%	*n*	*N*	%
Hypertension	109	206	52.9	99	199	49.7
Diabetes	72	200	36.0	80	199	40.2
Pre-existing diabetes	55	225	24.4	56	219	25.6
Newly diagnosed diabetes	13	225	5.8	11	219	5.0
Cardiac disease	13	188	6.9	14	186	7.5
COPD	2	187	1.1	12	186	6.5
Asthma	13	189	6.9	7	188	3.7
Chronic renal failure	18	189	9.5	14	186	7.5
Malignancy	8	184	4.3	4	183	2.2
TB active	1	174	0.6	4	169	2.4
TB past	4	101	4.0	9	107	8.4
HIV-positive	26	123	21.1	28	100	28.0

HIV, human immunodeficiency virus; COPD, chronic obstructive pulmonary disease; TB, tuberculosis.

A total of 159 patients (36%) hospitalised with COVID-19 died (82 male [51.6%] and 77 female [48.4%] patients) (*p* = 0.48) (78 elderly patients [49.1%] and 81 adults [50.9%]). No death occurred in the adolescent group (Table 2). A total of 392 patients with COVID-19 were admitted to the general ward and 52 persons required admission to an advanced level of care (i.e. high care and ICU, respectively). The majority of patients who were hospitalised and admitted to the general ward, required supplemental oxygenation (84.2%). However, only 31 (7%) patients required mechanical ventilation in the ICU. Given the high number of patients who were admitted (*N* = 444), more than 31 patients surely needed escalation to ICU (Table 3). However, on a multivariable analysis, ICU admission was an independent risk factor for mortality (Table 4). The ICU admission did not improve the odds of patient survival.

**TABLE 2:** Univariate analyses of risk factors and COVID-19 outcomes.

Variables	Outcome						*p*-value
	Alive			Died			
	*n*	*N*	%	*n*	*N*	%
**Gender**	-	285	-	-	159	-	0.4792
Female	148	-	51.9	77		48.4	-
Male	137	-	48.1	82		51.6	-
Age, median (IQR)	53.0	-	39.0–63.0	64.0		52.0–74.0	< 0.0001
**Age groups (years)**	-	285		-	159		< 0.0001
12–17	5	-	1.8	0	-	0.0	-
18–34	42	-	14.7	14	-	8.8	-
35–49	72	-	25.3	21	-	13.2	-
50–64	105	-	36.8	46	-	28.9	-
65 and above	61	-	21.4	78	-	49.1	-
**Hypertension**	120	255	47.1	88	150	58.7	0.0240
**Diabetes**	84	253	33.2	68	146	46.6	0.0081
**Diabetes status**							0.5345
Pre-existing	57	71	80.3	54	64	84.4	-
Newly diagnosed	14	71	19.7	10	64	15.6	-
**Cardiac disease**	16	235	6.8	11	139	7.9	0.6898
Chronic obstructive pulmonary disease	9	231	4.8	5	142	3.5	0.8532
**Asthma**	16	237	6.8	4	140	2.9	0.1031
**Chronic renal failure**	19	235	8.1	13	140	9.3	0.6873
**Malignancy**	5	226	2.2	7	141	5.0	0.1493
**TB active**	2	215	0.9	3	128	2.3	0.2908
**TB past**	6	140	4.3	7	68	10.3	0.0931
**HIV-positive**	33	152	21.7	21	71	29.6	0.2014
**Smoking**							0.5831
Never smoked	97	117	82.9	67	82	81.7	-
Current smoker	6	117	5.1	7	82	8.5	-
Previous smoker	14	117	12.0	8	82	9.8	-
**Obesity**	32	107	29.9	29	85	34.1	0.5336
**Intensive care unit admission**	14	285	4.9	35	159	22.0	0.0000
**Length of hospitalisation, median (IQR)**	9.0	-	5.0–14.0	4.0	-	2.0–9.0	< 0.0001

TB, tuberculosis; HIV, human immunodeficiency virus; IQR, interquartile range.

**TABLE 3:** Intervention and setting of care.

Intervention	*n*	%
**Supplemental oxygen**	374	84.2
**Mechanical ventilation**	31	7.0
**Ward setting**		
General ward	392	88.3
High care	3	0.7
Intensive care unit	49	11.0

**TABLE 4:** Logistic regression analyses using the step-wise method.

Variables	Odds ratio	95% CI	Estimate	Standard error	Wald stat	*p*-value
Age	1.044	1.029–1.062	0.04433	0.008170	29.44	< 0.0001
Length of hospital stay	0.923	0.892–0.955	−0.08036	0.017497	21.09	< 0.0001
ICU admission	3.235	2.099–4.989	1.17389	0.220665	28.30	< 0.0001
Hypertension	1.022	0.788–1.326	0.02213	0.132557	0.03	0.8674
Diabetes	0.854	0.664–1.100	−0.15758	0.128869	1.50	0.2214

CI, confidence interval; ICU, intensive care unit.

In terms of comorbidities present, only hypertension and diabetes (*p* = 0.0240 and 0.0081, respectively) were significantly related to death on univariate analysis (Table 2). On multivariable analysis, however, hypertension and diabetes were no longer significant (Table 4). Communicable diseases, such as active tuberculosis (TB) and HIV, with only five and 54 cases reported, respectively, had no significant impact on survival (*p* = 0.291 and *p* = 0.201). Regarding respiratory illnesses, COPD and asthma collectively, fared better as they had more survival than deaths (Table 2). Smoking, as a risk factor, did not seem to contribute much negatively to the adverse outcome as expected, probably because of lesser individuals self-reporting to be current or previous smokers.

**FIGURE 3:**
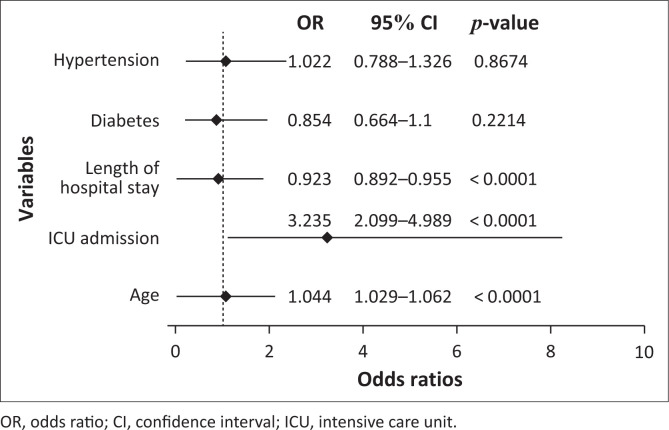
Variables and odds ratios for death in a logistic regression model.

Using the logistic regression model, the two predictor variables, age and ICU admission were significantly associated with the odds of non-survival. Older age was a significant risk factor for COVID-19-related death. The odds of dying increased by 1.044-fold for every additional year of age (odds ratio [OR]: 1.044; 95% CI: 1.029–1.062). Being admitted to the ICU was an independent predictor of mortality, which increased the odds of non-survival three-fold (OR: 3.325, 95% CI: 2.099–4.989). The average length of hospitalisation to reach an endpoint of non-survival was 4 days (Figure 3 and Table 4).

## Discussion

Comorbidities predispose patients with COVID-19 infection to worse clinical outcomes.^1,7,9,10^ Naturally, not every pre-existing comorbidity has the same dangers. For instance, in this global outbreak, the three most significant pre-existing conditions, namely hypertension, cardiovascular disease and diabetes were primarily linked to illness severity and unfavourable outcomes.^4^ However, in our study, hypertension and diabetes were not independent predictors of the adverse outcome, only age and ICU admission were. This differed with a trend observed in studies from China and the United States (US).^9,11^ The missing data might have had an impact on multivariable analysis. Tuberculosis and HIV seemingly had no negative impact on the outcome, despite available data that showed their prevalence.^10,12,13^ The plausible explanation could be because of under-investigation of these conditions during the pandemic because of the lockdown restrictions and staff shortage in health facilities. This created a barrier for healthcare access and led to reduction in HIV and TB testing.^14^

In certain studies, being ≥ 60 years of age was associated with an increased risk of disease severity.^1,15,16^ Furthermore, we demonstrated increased risk of death for each additional year of age (OR: 1.044; 95% CI: 1.029–1.062). The OR below one was considered a protective factor. The majority of patients treated throughout the study period were adults (between 50 years and 64 years old) and elders (65 years and above) with coexisting disorders, increasing their risk of developing severe disease. In South Africa, the second wave of COVID-19 saw increased hospitalisation of patients between 40 years and 64 years and above 65 years.^10^ The study underscored the importance of capacitating the healthcare service in order to protect the most vulnerable groups of society such as the elderly population. Vaccination against COVID-19 infection in older people offers a more significant protection. It had been shown to reduce hospitalisation and mortality.^17^

In our study, 49 patients (11%) required ICU admission, with 31 (7%) requiring mechanical ventilation. As compared to the data emanating from the US, one study reported that 809 (14.2%) patients received ICU care, with 695 (12.2%) of them receiving mechanical ventilation.^9^ The disparity in terms of available access to advanced care cannot be ignored. Our study was carried out in a setting with limited resources as compared to the US study. In the African COVID-19 Critical Care Outcomes study (ACCCOS), a combined 3752 (55.3%) out of 6779 patients from 64 centres in 10 countries were admitted to ICU. The 30-day in-hospital mortality was 48.2%.^16^

Our in-hospital mortality was 36%. This was associated with a shorter length of hospitalisation, with an average of 4 days. This could be because of severe disease on admission, late patient presentation or delay in receiving urgent medical care as a result of an overwhelmed healthcare facility. In comparison, a regional hospital in Mthatha, Eastern Cape province, which also serves a similar mainly rural population as Pietersburg Hospital, had 46% mortality rate, way above the provincial rate (27.5%) and national rate (18.3%).^18^ In this study, a high volume of patients coupled with a lack of resources and late presentation contributed largely to the high in-hospital mortality.^18^ In a large, geographically inclusive retrospective study performed in the US, the in-hospital mortality rate was 10.6% in March 2020 with an increased trend towards 19.7% in April 2020, declining to 9.3% in November 2020. This improvement was largely because of better understanding of the disease, improved clinical care and ventilation strategies.^19^ South Africa experienced more in-patient mortality during the second wave. This was driven largely by the new Beta variant that peaked in January 2021, coupled with increased admissions of older patients and increased healthcare facility load.^10^ The Limpopo province was also hard-hit, with 90% deaths reported during the second wave. The case fatality rate was 27.5%.^20^

There was no significant association between non-survival and communicable diseases such as active TB and HIV, respiratory illnesses such as asthma and chronic obstructive pulmonary disease. This correlates with the findings by ACCCOS multicentre cohort study with the exception that in their case, HIV was associated with increased mortality in adults admitted with COVID-19.^16^ In our case, fewer patients reported having HIV or active TB. This could be attributed to the under-investigation of patients who were admitted. A larger, countrywide research is suggested on survivors of COVID-19 infection to further understand the long-term impact of the pandemic.

Obesity has been strongly associated with COVID-19 mortality in the literature.^5^ Even though it was associated with comorbidities including hypertension, diabetes, and cardiovascular illnesses, this was frequently linked to reduced lung capacity exacerbated by viral and secondary pneumonia.^5^ In our investigation, obesity was determined based on the clinician’s subjective assessment as the standard method of calculating body mass index (BMI) was not objectively used. This might have been because the COVID-19 infection was so severe that the majority of patients became oxygen dependent, making it challenging to measure objectively. Another explanation could be that even individuals with mild COVID-19 disease were not properly identified because calculating BMI is not a typical practice in our setting. Therefore, in our study, the effect of obesity was not statistically significant and we acknowledge the limitation that obesity was not objectively measured, hence the result should be taken with caution.

### Study limitations and strengths

This was a retrospective study; therefore, limitations are unavoidable. The collection of data relied mostly on healthcare personnel to enter data onto the portal; therefore, some pertinent information might have not been captured. Data were from a convenient sample of hospitalised adult patients from a single centre, not a representation of a whole population. Patients were not followed up post discharge in this study, hence the study outcome might have been under-reported as some patients who were discharged or transferred to other facilities could have suffered the adverse events thereafter. As noted above, obesity was not objectively measured and together with underestimation of TB and HIV, could have influenced the association with outcomes. However, the strength of the study is that it utilised a large sample size.

## Conclusion

According to the study, the risk factors that were independently associated with in-hospital mortality were older age and ICU admission. Comorbidities did not contribute significantly to the outcome in this study, as reported elsewhere.^9,11^
